# Inflammation and Parkinson's Disease

**DOI:** 10.4061/2011/729054

**Published:** 2011-12-28

**Authors:** Carlos Barcia, Stéphane Hunot, Gilles J. Guillemin, Fernando Pitossi

**Affiliations:** ^1^Clinical and Experimental Neuroscience, CIBERNED, Department of Human Anatomy and Psychobiology, School of Medicine, University of Murcia, Campus de Espinardo, 30100 Murcia, Spain; ^2^CRICM, INSERM/UPMC UMR-S975 (Ex-U679), CNRS UMR 7225, Experimental Therapeutics on Neurodegeneration, Salpetriere Hospital, 47 Boulevard de l'Hôpital, 75013 Paris, France; ^3^Department of Pharmacology, The University of New South Wales, Wallace Wurth Building, Sydney, NSW 2052, Australia; ^4^Instituto Leloir, IIBBA-CONICET, Avenue Patricias Argentinas 435, 1405 Buenos Aires, Argentina

After more than two decades of research, the latest published studies regarding the protective effects of anti-inflammatory drugs in Parkinson's disease (PD) indicate that only a subset of nonsteroidal anti-inflammatory drugs (NSAIDs) may be efficient in decreasing the risk of PD. In particular, recent epidemiology studies and meta-analysis have shown that, among these NSAIDS, the inhibitors of the enzyme cyclooxygenase (COX) were the most potent compounds reaching the highest rate of PD prevention. These data clearly support a COX-specific mechanism of neuroprotection and reinforce the idea that neuroinflammation in PD comprises specific features that should be unraveled. Such that knowledge should help the development of specific drugs targeting inflammatory mediators.

Several clinical trials currently ongoing have focused their goals in evaluating *in vivo* potential imaging biomarkers for inflammatory changes in neurodegeneration. [18F] FEPPA and [(11) C] PBR28 are being evaluated for their capacity in detecting neuroinflammation by single photon emission computed tomography (SPECT). This is an important step regarding the safe monitoring of neuroinflammation in patients. If successful, these *in vivo* imaging methods will be valuable not only to determine precisely the right therapeutic window but also to accurately measure the biological outcomes of neuroprotective treatments. Most importantly, this also means that the inflammatory component of PD is having significant attention among researchers and it will probably be assumed in the clinical scenario in the coming years. In addition, there are numerous preclinical trials testing the protective effects of anti-inflammatory drugs in animal models of PD and hopefully some of them will soon be brought into Phase I trials. However, further research and new perspectives are needed to understand the specific aspects of inflammation in PD.

In the present special issue, we present 9 review articles that explore new insights into the inflammatory reaction associated with PD. D. Litteljohn et al. show an excellent review of how the toxin-based models of PD have contributed significantly to the study of the mechanisms underlying neuroinflammatory processes in Parkinsonism and outline the role of TNF-*α* and IFN-*γ*, two cytokines critically involved in glial cell activation and dopaminergic degeneration. Then, T. Farooqui and A. A. Farooqui describe in a comprehensive review how lipid-derived factors are able to induce cellular stress and inflammation, which may be involved in PD pathogenesis. In line with this, M. Liu and G. Bing show in their revision how lipopolysaccharide (LPS), a cell-wall component of Gram-negative bacteria and prototypical inflammogen, induces dopaminergic cell death indicating that the inflammatory response is by itself detrimental. Importantly, inflammation-induced toxicity seems to be highly specific for dopaminergic cells and with very special distinctiveness in some dopaminergic areas. In fact, V. Roca et al. nicely described, in their review, the unique susceptibility to inflammation of the Substantia Nigra, the prime locus of dopaminergic cell death in PD.

On the other hand, C. C. Ferrari and R. Tarelli describe in their complete study how systemic inflammation may impact central inflammation and dopaminergic cell death. Then, they explore the possibility that peripheral inflammation could be a contributing factor in PD development. This view is further complemented by the review from A. Machado et al., which gives a different perspective of this particular topic but focused on studies performed in animal models of PD.

Finally, in a last series of review, authors consider new perspectives and therapeutic targets to avert toxic inflammation in PD. First, A. R. Carta et al. discuss an emerging strategy to block neuroinflammation in PD using PPAR-*γ* agonists. This is particularly interesting in light of the above discussion since ibuprofen but not aspirin or acetaminophen has been shown to display PPAR-*γ* agonistic activity. P. M. Flood et al. proposed new ways for targeting NF-kB through IKK complex inhibition, a promising route that may be a useful approach in a close future in the treatment of PD. Finally, in a last review article, A. Zinger et al. describe how the kynurenine pathway, a metabolic pathway involved in the production of nicotinamide adenine dinucleotide, may be critically involved in the neuroinflammatory process in PD and how this pathway could be controlled for therapeutic purposes.

*Carlos Barcia* *Carlos Barcia* *Stéphane Hunot* *Stéphane Hunot* *Gilles J. Guillemin* *Gilles J. Guillemin* *Fernando Pitossi* *Fernando Pitossi*

